# Expectations, values, preferences and experiences of Hungarian primary care population when accessing services: Evaluation of the patient’s questionnaires of the international QUALICOPC study

**DOI:** 10.1017/S1463423620000596

**Published:** 2021-06-01

**Authors:** Anna Nánási, Tímea Ungvári, László R. Kolozsvári, Szilvia Harsányi, Zoltán Jancsó, Levente I. Lánczi, Lajos Mester, Csaba Móczár, Csilla Semanova, Péter Schmidt, Judit Szidor, Péter Torzsa, Mária Végh, Imre Rurik

**Affiliations:** 1 Department of Family and Occupational Medicine, Faculty of Public Health, University of Debrecen, Debrecen, Hungary; 2 Doctoral School of Health Science, University of Debrecen, Debrecen, Hungary; 3 Institute of Family Medicine, Faculty of Medicine, University of Szeged, Szeged, Hungary; 4 Irinyi Primary Care Health Center, Kecskemét, Hungary; 5 Department of Family Practice, Faculty of Medicine, Semmelweis University, Budapest, Hungary; 6 Institute of Primary Care, Faculty of Medicine, University of Pécs, Pécs, Hungary

**Keywords:** adherence, expectations, Hungary, preferences, primary care, QUALICOPC

## Abstract

**Background:**

Preferences and wishes of patients is an important indicator of primary health care provision, although there are differences between national primary care systems.

**Aim:**

The aim of this paper is to describe and evaluate the preferences and values of Hungarian primary care (PC) patients before accessing and to analyse their experiences after attending PC services.

**Methods:**

In the Hungarian arm of the European *QUALICOPC Study*, in 2013–2014, information was collected with questionnaires; the *Patient Values* contained 19 and the *Patient Experiences* had 41 multiple-choice questions.

**Findings:**

The questionnaires were filled by 2149 (840 men, 1309 women) using PC services, aged 49.1 (SD ± 16.7) years, 73% of them having chronic morbidities. Women preferred to be accompanied and rated their own health better. Patients in the lowest educational category and women visited their GPs more often, and they are consulted more frequently by other doctors as well. Men, older and secondary educated people reported more frequently chronic morbidities. Longer opening hours were preferred by patients with higher education. The most preferred expectations were availability and polite communication of doctors, not pressures on consultation time, clear instructions provided during consultations, shared decisions about treatments and options for consultations, the knowledge of the doctors concerning the living conditions, social and cultural backgrounds of patients, updated medical records, short waiting times, options for home visits, wide scope of professional competences and trust in the doctor.

**Conclusion:**

Wishes, preferences of patients and fulfilment were similar than described in other participating countries of the study. Although there are room to improve PC services, most of the questioned population were satisfied with the provision.

## Introduction

Many studies proved that in countries where primary care (PC) system is stronger, the healthcare system performs better (Macinko *et al.*, 2003). Strong PC has to response to the patients’ needs, expectations and preferences as well (Schäfer *et al.*, 2011). There is a big variation between individuals, therefore at the patients’ level as well. What do patients expect the general practitioners (GPs) to take within the consultation and to what extent are these expectations fulfilled? What factors influence the expectations of the patients and the actions of GPs? (Webb and Lloyd, 1994). Why and when do patients visit doctors? They could have been different influence on daily activities and symptom burden, such as the total number of symptoms experienced by each person (Elnegaard *et al.*, 2017).

In 2010, the three-year Quality and Costs of Primary Care in Europe (QUALICOPC) study was planned, aiming to compare and analyse how the primary health care systems of 35 countries perform in terms of quality, cost and equity. The study analysed three levels of PC. The *service provision* level, covering characteristics of the GP practice, *organisation* and the *type of services* that are delivered and the *patient* level, where the users of services experience whether the care provided responds to their needs and expectations (Schäfer *et al.*, 2013).

Family physicians/GPs were chosen as one of the survey subjects. Beside GPs, their patients were also approached and questioned using 2 other questionnaires, to explore their expectations before and their experiences after using the services.

The aim of this paper was to describe and evaluate the expectations, personal values and experiences of Hungarian people who attended to PC services, based on the information collected within the Hungarian arm of the *QUALICOPC Study*, using two questionnaires. Two questionnaires, developed by the QUALICOPC researchers, were used. In each participating country, the response target was 220 GPs and 2200 patients (10 per each).

The questionnaires were translated in the respective national language(s) via an official forward- and back-translation procedure. The *Patient Values questionnaire* contained 19 questions (statements with multiple choice answers), four questions focused on communication between GPs and patients. Both questionnaires were previously tested and validated (Schäfer *et al.*, 2013).

The *Patient Experiences questionnaire* included 41 multiple choice questions, asking to what extent the patient agrees with the statement given. There were questions on the patient’s background and socio-economic status, perceived constructed for patients.

## Method

### Structuring the questionnaires, study design

Health, reason for visiting the GP, and visits to medical specialists and hospitals, experiences with ‘*continuity of care’*, use of medical records and time slot, available for patient. *Quality of care* as experienced by patients, *accessibility of care*, divided into physical and financial access. There were inquiries on home visits and waiting times, towards *equity in access* and *equity in treatment*, experiences of coordination in the case of referral, on treatment by a practice nurse, about patient’s involvement in decision making and referrals, beside their adherence to the treatment plan. *Comprehensiveness of services* offered by the GP was also probed in a question about patients’ views on the breadth of the clinical task profile of services.

### Distributing questionnaires, settings

The study centre of the Hungarian arm of QUALICOPC project was established at the University of Debrecen, with close cooperation with the other Departments of Family Medicine (Budapest, Pécs, Szeged). An advertisement was issued to recruit participating GPs in the whole country. Two hundred-twenty two GPs who wanted to participate were selected randomly, based on the order of application. Population density and expected geographically representativeness were also considered (Rurik *et al.*, 2012).

During the study period (2012–2014), the questionnaires were transported to the practices by educated fieldworkers, who were usually medical students. They gave one questionnaire per practice, to the nearest patients in the waiting room (*Patients’ value*) and contacted nine other patients consecutively, who left the surgery to summarise (*Patients Experiences*).

### Presentation of data

The original order of questions was followed. There were 12 identical questions in the questionnaires; therefore, the overlapped answers were presented together. Distributions are always presented and statistical correlations, when found. In some columns, similar answers were merged. Options, with only a few number of responses were missed.

Statistical analyses were performed with STATA software.

### Ethics

The Hungarian Research Ethical Committee in Medicine (TUKEB) approved the study assigned the number: 20024/2011-EKU (643/PI/11.).

## Results

The *Patient Values questionnaire* was filled by 214 persons (139 men, 75 women). Their mean age was 47.2 years (SD ± 17.6).

Younger, more educated persons and women were satisfied better with their health status, when describing their own health in general. Men, older and secondary educated people reported more frequently chronic morbidities.

The *Patient Experiences questionnaire* was filled by 1935 persons; men: 701 (36%), women: 1234 (64%). Their mean age was 49.6 (SD ± 16.7) years.

Answers options *important* and *very important* were merged into one column in Table 1.

**Table 1.** The experiences and expectations of patients regarding circumstances, services, provided information, behaviour and consultation’s skills of family physicians

How important are the following to you (*n* = 2149)	Important +Very Important [%]
That I understand clearly what this doctor explains	90.7
That people at the reception desk are polite and helpful	86.0
That I feel able to cope better with my health problem/illness after this visit	81.4
That this practice has extensive opening hours	71.6
That I can get an appointment easily at this practice	75.4
That this practice is close to where I live or work	79.1
That I have a short waiting time on the phone when I call this practice	84.7
That I don’t need to tell a receptionist or nurse about details of my health problem before seeing my doctor	59.5
That the doctor has prepared for the consultation by reading my medical notes (# it was rated as the most important)	#73.5
That I have prepared for the consultation by keeping a symptom diary or preparing questions	66.5
That I can bring a family member/friend to the consultation if I think this is useful	58.6
That I know which doctor I will see	80.0
That I keep to my appointment	80.5
**During the consultation**	
That the doctor makes me feel welcome by making eye contact	82.8
That the doctor listens attentively	95.4
That the doctor does not give me the feeling to be under time pressure	89.3
That the doctor is aware of my personal. social and cultural background	71.2
That the doctor is not prejudiced because of my age, gender. religion or cultural background (# it was rated as the most important)	#82.3
That the doctor treats me as a person and not just as a medical problem	91.2
That the doctor is respectful during physical examination and by not interrupting me	86.5
That the doctor takes me seriously	93.5
That the doctor understands me	86.5
That the doctor asks me if I have any	81.9
That the doctor asks if I have understood everything	83.7
That the doctor knows when to refer me to a medical specialist	83.3
That the doctor avoids disturbances of the consultation by telephone calls etc.	72.6
That the doctor gives me additional information about my health problem e.g. leaflets	59.1
That the doctor informs me about reliable sources of information e.g. websites	43.7
That I tell the doctor what I want to discuss in this consultation	68.8
That I am prepared to ask questions and take notes	36.3
That I am honest and not feel embarrassed to talk about my health problem	76.8
That I am open about my use of other treatments. such as self-medication or alternative medicine	44.7
That psychosocial issues (for example personal worries) can be discussed if needed	52.6
**After the consultation**	
That the doctor gives me all test results. even if they show no abnormalities	78.1
That the doctor offers me to have telephone or email contact if I have further questions	60.0
That the doctor gives me clear instructions on what to do when things go wrong (# it was rated as the most important)	#92.1
That I adhere to the agreed treatment plan	89.3
That I inform the doctor how the treatment works out	86.5
That I can see another doctor if I think it is necessary	71.6

Almost all participants of the two surveys (97.2%) and their mothers (96.3%) were born in Hungary. In the same household, 77.2% lived with adult family members and 33.4% with children under 18 years of age.

Regarding employment status, 37% worked in civil service, 8% as self-employed, 29% retired and 7% student, 8–8% were disabled and unemployed, 52 % estimated their income around and 42.6% below the average.

Women patients preferred significantly (*P* = 0.007) better to be accompanied by family members to the consultation, and according to their reports, they could cope better with health problems after the visit (*P* = 0.071). Longer opening was preferred better (*P* = 0.035) by patients with higher education.

Majority of patients (84.1%) visited their own, registered family physician. Presence of chronic or longstanding conditions (high blood pressure, diabetes, depression, asthma, etc.), description of own health in general, frequency of consultation with GPs in the last 6 months and consultations with specialist in the previous year are presented in the figures of Table 2.

**Table 2.** Rating own health, presence of chronic condition, frequency of visits by GPs and consultation with specialist, according to age cohort, gender and educational level [percent]

Age cohort				Education/qualification			Gender	
Own health	<35 years	35–60 years	>60 years	no	Post-secondary	Upper secondary	Women	Men
Fair	56.3	55.2	34.1	48.1	37.5	44.8	45.7	41.9
Good	17.2	18.4	46.7	15.9	43.7	36.3	33.5	31.9
Very good	1.6	1.4	13	5.1	13.1	6.2	5.4	10.9
Poor	25	25.0	6.3	31	5.6	12.7	15.5	15.4
**Chronic condition**	76.9	79.4	32.4	73.7	43.2	52.1	54.4	55.8
**Frequency of visits in the last 6 months by GP**								
5 times or more before	40.9	42.6	18.0	45.1	18.9	27.6	31.6	27.5
2 to 4 times before	42.4	36.9	33.2	30.5	37.5	36.0	36.3	33.0
Once before this visit	7.6	11.7	25.0	11.5	23.1	19.8	17.3	20.3
This was the first visit	7.6	6.5	19.6	7.8	17.6	13.8	11.5	15.8
**Frequency of consultations with medical specialist in the past 12 months**								
None	0.0	5.3	11.4	8.2	5.9	8.7	7.7	9.6
Once or twice	17.2	23.3	39.6	21.3	35.6	33.7	30.4	33.4
3 to 5 times	23.4	28.3	29.7	26.3	35.2	27.9	29.5	27.8
6 to 10 times	23.4	21.7	9.1	20.6	11.3	14.6	15.3	14.8
More than 10 times	35.9	21.3	10.3	23.6	11.8	15.1	17.0	14.5

*n* = 1935.

Women rated their own health to be better. Logistic regression analysis was performed, for gender: correlation coefficient: 0.18, standard error: 0.3, *P* < 0.001 and 95% confidence interval: [0.11; 12:24].

Patients in the lowest educational category visited more often their GPs, females consulted more frequently, proved by logistic regression analysis. For gender, correlation coefficient: 0.13, standard error: 0.04, *P* < 0.001 and 95% confidence interval: [0.06; 0.20].

The main reason for actual practice visit was a recent illness (30.7%), medical check-up (24.4%), to get prescription (42.9%) or referral (9.8%), second opinion (12.4%), asking a medical certificate (6.9%). Other reason was mentioned by 16.7%.

Experiences regarding the actual visit, content of consultation and agreement about the listed statements are described in Table 3. Doctors dealing with not medical problems only, giving more attention to personal problems and worries, were preferred better by patients with higher education (*P* = 0.01) and by women (*P* = 0.002). Listening carefully to the patients was requested better by women as well (*P* = 0.08). In 71.8% of the cases, the time of travel between the home and the GP’s office was less than 20 minutes. Twenty one percent of patients made an appointment, 85% of them got it easy, 29% made it the same day, 37% a day before, while 19% had to wait for 2–7 days. One third of patients had to wait less than 15 minutes, 29 % waited 15–30 minutes.

**Table 3.** Statements and opinion about the doctor, experiences regarding the actual visit, content of consultation and agreement about the listed statements [percent]

Do you agree with the following?	Yes [%]
The doctor had my medical records at hand	78.9
The doctor was polite	96.8
The doctor listened carefully to me	95.1
The doctor asked questions about my health problem	90.9
I couldn’t really understand what the doctor was trying to explain	20.5
The doctor took sufficient time	92.9
The doctor involved me in making decisions about treatment	84.3
I would recommend this doctor to a friend or relative	89.0
The doctor asked about possible other problems besides the one I just came for	64.8
He/she knows important information about my medical background	85.1
He/ she knows about my living situation	63.1
This doctor doesn’t just deal with medical problems but can also help with personal problems and worries	42.9
After this visit, I feel I can cope better with my health problem/ illness than before	62.7
In the past 12 months, has a GP from this practice talked to you about how to stay healthy? (For instance about diet, alcohol or smoking)	65.9
In past 2 years, has a GP from this practice ever asked you about all the medications you take (also those prescribed by other doctors)?	79.5
If I need a home visit I can get one	79.0
I know how to get evening, night and weekend services	73.0
People were polite and helpful at the reception desk	91.9

*n* = 1935.

Negative experiences of patients were listed in Table 4. Most of the patients were informed that there is an option to change their doctors, if not satisfied with manner or services.

**Table 4.** Negative experiences and feelings of patients

In the past 12 months, has one of the following happened to you in this practice? (*n* = 1935)	Yes (%)
The doctor or staff acted negatively to you	4.0
Other patients were treated better than you	3.1
The opening hours are too restricted	15.8
The doctor was too much concerned about money	2.2
It is too difficult to see a GP during evenings, nights and weekends	10.9
The doctor or staff showed disrespect because of your ethnic background	3.5
The doctor or staff showed disrespect because of your gender	4.1
I thought tests or examinations were repeated unnecessarily	2.5
I thought I got the wrong medication or wrong dose	3.7
I thought I got incorrect results of a test or X-ray	2.1
If you are unhappy with the treatment you received, do you think this doctor would be prepared to discuss it with you?	82.6

Within the whole study population, 507 persons did postpone or abstain from a visit to the GP in the past 12 months, despite they needed it. Forty four percent of the patients had to cancel their planned visits because she/he was too busy, 11.6% could not get there (physically). Financial reasons were mentioned by 12.8% and only 2.8% did not have insurance. Other reasons for missed visits were 34.7%.

In the case of consultations, 84% of patients believed that their GP was informed about the finding, 61% stated that specialist was informed by the GP appropriately and only 7% experienced difficulties during referral.

Six hundred fifty of the interviewed persons had personal experiences about using *out of hour* services or emergency departments. The most frequent reasons for encounter were morbidities or complain out of the scope of GP (46.5%), out of the opening time of GPs (21.7%), 5.5% expected a shorter waiting time, 6.8% mentioned that emergency department is more convenient to reach.

The preferences and expectations of patients with complaints, in the case of the listed symptoms are described in Table 5.

**Table 5.** Preferences and request for GP services in presence of the listed symptoms [percent]

How important would it be for you to see a doctor if you had (*n* = 1935)	Extremely important + rather important [%]
Weight loss of more than 2 kg in a month when not dieting	42.7
Shortness of breath with light exercise or light work	62.5
Chest pain when exercising	77.9
Loss of consciousness, fainting or passing out	91.7
Headache for more than one day	49.2
Abdominal pain for more than one day	54.2
Severe worries for more than a month	64.1
Do you expect to benefit from a GP visit for ….	Yes [%]
Stomach problems	83.8
Shoulder and neck pain	73.9
Feeling nervous	43.7
Diarrhoea	69.1
Sore throat	69.6
Headache	49.3
Feeling tired	40.5
Flu	53.5
Feeling nauseous	51.0
Would most people visit a GP for the following?	Yes (%)
Cut finger that needs to be stitched	18.5
Removal of a wart	11.6
Routine health checks	73.3
Deteriorated vision	33.0
Help to quit smoking	39.3
A child with a severe cough	66.8
Stomach pain	77.4
Blood in the stool	76.1
Sprained ankle	36.5
Anxiety	43.6
Domestic violence	15.2
Sexual problems	11.7
Relationship problems	8.0
Advice for choosing the best hospital or specialist for a certain treatment	61.6

Only 22.8% of patients were examined or treated by a nurse in the GP’s practice. Patients have a great confidence to their GPs. The statement ‘*In general, doctors can be trusted*’ were strongly agree by 33.5%, simple agree by 61.3% of the questioned persons

## Discussion

### Main findings

Patients’ expectations are mainly focusing on professionalism, comfort and accessibility of services.

In *professional* term: updated knowledge and good manners of doctors, wide scope of complaints to be able to solve, easy to get prescriptions, no barriers to referrals, common decision making about treatment, in respect of the clinical outcomes and also the emotional and human features of the consultation are the highlights of the patient’s expectations.

Preferences regarding circumstances, facilities, courteous communication, clear instructions, adequate information about living circumstances, social and cultural background of the patients were mentioned as well.

Easy *access* to services, availability and short waiting time, option for home visit, not pressured by time during consultations are also expected.

### Limitations

Our study was focused only to the Hungarian characteristics; answers of the patients about their preferences and experiences should be evaluated by taking into consideration specific national traits and a variety of PC provision depending strongly from the personality and available infrastructure of the physicians.

It is not sure that questioned persons are representative in social and economic points of view.

After translating and launching the questionnaire, there was no option to clarify questions having different meanings in different countries.

Being part of an international study, we had to follow the original protocol, recruitments and presentations of the findings.

Before and after this study no such a survey was performed in Hungary. Since structure and utilisation of PC did no change in the past years, these findings could be valid nowadays as well.

There are no financial or administrative restrictions on the availability of PC services in Hungary. It can be used by all citizens, although a social insurance ID card is required before enrolment into a practice.

Hungary is relatively a closed country, hence almost all of the participants (also their mothers) were native Hungarians, and only small percent belonged to ethnic minority. According to the effective legislations in Hungary, it is strictly forbidden to register ethnic or national origin in any medical or official files. In the neighbouring Slovenia, where 6.5% of the PC population are migrants, often experiencing negative attitude from GPs (Jakič and Rotar Pavlič, 2016).

Walk-in accident and emergency services have been established in the Hungarian hospitals only in the last two decades; patients tend to visit them only if PC services are not available.

In the Hungarian primary care, there are no traditions of appointments; patients were served by the order of arrival. The ratio of appointments is continuously increasing due to the order of the Minister of Health. These scheduled services are becoming increasingly popular.

The time waiting for appointment is usually longer in Canada (Premji *et al.*, 2018) and in the Nordic countries (Tolvanen *et al.*, 2018), especially for older patients.

There were only small differences between expectations of different age groups; older patients were more satisfied with the care, perhaps their expectations were lower (Bowling *et al.*, 2013). Higher scores of experience may not illustrate better consultations as such; it is the lower levels of initial expectations that determine the level of patient satisfaction (Ogden and Jain, 2005). The results revealed that patients with greater numbers of their expectations met reported significantly higher satisfaction with the consultation than those with lower numbers met (Williams *et al.*, 1995). Generally, GP patients reported higher pre-visit expectations and post-visit met expectations, reflecting chiefly doctor-patient communication style and the doctor’s approach to providing detailed information (Bowling *et al.*, 2012).

Referrals of patients from the primary to specialist care are important in all health care system. Patients were most positive if the physician had initiated the referral, which supports the gate-keeper role of the GP (Rosemann *et al.*, 2006). As gate-keeping is very weak in Hungary, the preferences of patients are mostly respected. Some specialists could be accessed without referral. Obtaining a letter of referral is often the reason why GPs are contacted; the referrals to specialist are often requested by patients, mainly in the bigger cities. The preferences and expectations of Hungarian patients were not always in agreement with their experiences and values. Findings in the literature regarding the relationship between strong PC and the responsiveness to patient expectations and needs are inconclusive (Ashworth and Armstrong, 2006). Patient satisfaction was found to be lower in countries where the access to specialist services was regulated through gate-keeping (Bensing *et al.*, 2011; De Maeseneer *et al.*, 2003; Schellevis *et al.*, 2005).

Not all of the PC patients need a medical check-up, regular prescriptions and some consultations are done by practice nurses (Cockburn and Pit, 1997). However, ‘nurse practitioners’ are not yet involved in the Hungarian primary care.

In Hungary, smaller surgical procedures are routinely performed in rural or remote GP’s offices, while in cities, GPs usually prefer referring to the surgeons. The available equipment are less advanced than in Nordic countries (Eide *et al.*, 2017).

Most of the professional reasons for encounters are expected to be managed by the GPs. Patients prefer to visit their own GPs because all of their health-related information is available there, while computerised data are not always available in other countries (Lionis *et al.*, 2017).

Group practices do not yet exist in Hungary. The patient has a right to choose a GP, and GPs are obliged to accept all enrollers in the geographical area they cover. Patients usually visit their own GP in a single-handed practice. Differences in access between different practice models, like in Canada, do not exist in Hungary (Miedema *et al.*, 2016).

In bigger villages and cities, PC offices are easy to approach. Positive behaviour of doctors is well accepted, including consultation’s skills and manner. Like in other countries, majority of patients felt better able to cope with their health-related problem after an appointment with GP, reflecting patients’ enablement (Tolvanen *et al.*, 2017). Regarding communication between doctors and patients, no difference was proved, while it could be better in medium-sized practices (Eide *et al.*, 2016).

Unfortunately, preventive services are not appropriately implemented in the Hungarian primary care; the visits to doctors are mostly caused by chronic morbidities or acute complaints (Sándor *et al.*, 2016).

Population expectancy is influenced by national traditions and previous experiences (Janka, 2017). Hungarian GPs are managing many social issues, including administrative tasks and for the past 60 years (including decades of Communism) they were considered as the only stable points in the health care, mainly in the years when ‘reforms’ were initiated in the health care system. In the future, more focus needed to person-centred care, to better involvement of patient in decision-making and appropriate delivery of preventative services (Lionis *et al.*, 2017). Patients require equity, accessibility and good quality of PC services (Oleszczyk *et al.*, 2017).

Reasons for visits, medical problems to be solved and individual expectations were similar in the recent publications of other participating countries (Eide *et al.*, 2016; 2017; Miedema *et al.*, 2016; Lionis *et al.*, 2017; Oleszczyk *et al.*, 2017; Tolvanen *et al.*, 2017). In Hungary and in most of the participating countries, the QUALICOPC study proved a high population satisfaction with the primary health care system (Lionis *et al.*, 2017; Oleszczyk *et al.*, 2017; Sanchez-Piedra *et al.*, 2017; Tolvanen *et al.*, 2017). We are still waiting for the findings of other countries where the study run.
